# On the feasibility of deep learning applications using raw mass spectrometry data

**DOI:** 10.1093/bioinformatics/btab311

**Published:** 2021-07-12

**Authors:** Joris Cadow, Matteo Manica, Roland Mathis, Roger R Reddel, Phillip J Robinson, Peter J Wild, Peter G Hains, Natasha Lucas, Qing Zhong, Tiannan Guo, Ruedi Aebersold, María Rodríguez Martínez

**Affiliations:** Cognitive Computing & Industry Solutions, IBM Research Europe - Zurich, Rueschlikon 8803, Switzerland; Cognitive Computing & Industry Solutions, IBM Research Europe - Zurich, Rueschlikon 8803, Switzerland; Cognitive Computing & Industry Solutions, IBM Research Europe - Zurich, Rueschlikon 8803, Switzerland; ProCan®, Children’s Medical Research Institute, Faculty of Medicine and Health, The University of Sydney, Westmead, NSW, Australia; ProCan®, Children’s Medical Research Institute, Faculty of Medicine and Health, The University of Sydney, Westmead, NSW, Australia; Dr. Senckenberg Institute of Pathology, University Hospital Frankfurt, Frankfurt am Main, Germany; ProCan®, Children’s Medical Research Institute, Faculty of Medicine and Health, The University of Sydney, Westmead, NSW, Australia; ProCan®, Children’s Medical Research Institute, Faculty of Medicine and Health, The University of Sydney, Westmead, NSW, Australia; ProCan®, Children’s Medical Research Institute, Faculty of Medicine and Health, The University of Sydney, Westmead, NSW, Australia; Institute of Basic Medical Sciences, School of Life Science, Westlake University, Hangzhou 310024, China; Institute of Molecular Systems Biology, Department of Biology, ETH Zurich, Zurich 8093, Switzerland; Cognitive Computing & Industry Solutions, IBM Research Europe - Zurich, Rueschlikon 8803, Switzerland

## Abstract

**Summary:**

In recent years, SWATH-MS has become the proteomic method of choice for data-independent–acquisition, as it enables high proteome coverage, accuracy and reproducibility. However, data analysis is convoluted and requires prior information and expert curation. Furthermore, as quantification is limited to a small set of peptides, potentially important biological information may be discarded. Here we demonstrate that deep learning can be used to learn discriminative features directly from raw MS data, eliminating hence the need of elaborate data processing pipelines. Using transfer learning to overcome sample sparsity, we exploit a collection of publicly available deep learning models already trained for the task of natural image classification. These models are used to produce feature vectors from each mass spectrometry (MS) raw image, which are later used as input for a classifier trained to distinguish tumor from normal prostate biopsies. Although the deep learning models were originally trained for a completely different classification task and no additional fine-tuning is performed on them, we achieve a highly remarkable classification performance of 0.876 AUC. We investigate different types of image preprocessing and encoding. We also investigate whether the inclusion of the secondary MS2 spectra improves the classification performance. Throughout all tested models, we use standard protein expression vectors as gold standards. Even with our naïve implementation, our results suggest that the application of deep learning and transfer learning techniques might pave the way to the broader usage of raw mass spectrometry data in real-time diagnosis.

**Availability and implementation:**

The open source code used to generate the results from MS images is available on GitHub: https://ibm.biz/mstransc. The data, including the MS images, their encodings, classification labels and results, can be accessed at the following link: https://ibm.ent.box.com/v/mstc-supplementary

**Supplementary information:**

Supplementary data are available at *Bioinformatics* online.

## 1 Introduction

Proteins participate in virtually every process in the cell, and are directly responsible for its observed phenotype. Their accurate identification and quantification can therefore enable the precise characterization of phenotypes. Proteins are most commonly analyzed by mass spectrometry (MS). Among the available mass spectrometry approaches, SWATH-MS (Sequential Window Acquisition of all THeoretical fragment ion spectra) has emerged as a technology that combines deep proteome coverage, high reproducibility and quantitative consistency and accuracy (Gillet *et al.*, 2012a). In a SWATH-MS measurement, all ionized peptides falling within a specified mass range are fragmented in a systematic and unbiased fashion using large precursor isolation windows (Ludwig *et al.*, 2018). Spectral profiles are then recorded for all ionized peptides and fragment ions thereof.

While the raw data acquisition is unbiased, peptide identification requires prior information about the fragment ion patterns and the retention time (RT) of all targeted peptide fragments, which are typically extracted from SWATH assay libraries (Guo et al., 2015). Uncertainties in peptide identification result in inaccurate protein quantification and potential protein mis-identification, especially as only a few peptides per protein are typically detected. Protein isoforms and peptide modifications further complicate computational workflows and exacerbate the variability observed across experiments and platforms. Indeed, while a recent benchmark of different SWATH-MS data processing tools highlighted the convergent identification and reliable quantification performance of all tools (Navarro et al., 2016), careful pre- and post-processing and parameter optimization were needed to achieve robustness in label-free quantitative proteomics. The complexity of current MS data analysis workflows is partly responsible for their slow translation into clinical practice, despite having been long-postulated to enable a huge clinical impact (Aebersold and Mann, 2003).

Contrasting the carefully designed and parameterized workflows commonly used for the analysis of SWATH-MS datasets, we investigate whether state-of-the-art deep learning models could enable the circumvention of protein quantification and the execution of certain predictive tasks directly on raw mass spectrometry data.

*Deep learning* (DL) has become one of the most active fields in artificial intelligence, with spectacular performances in a broad area of applications such as computer vision, speech recognition and natural language processing. In parallel, recent years have witnessed an exponential increase in the number of DL applications in computational biology (Ching *et al.*, 2018). These works have demonstrated the extraordinary capacity of DL models to automatically learn discriminative features from raw data, thus eliminating the need for intricate feature-engineering. In contrast to targeted proteomic analysis, deep learning is particularly adept at learning abstract features directly from the raw data, with different layers of the network sequentially learning increasingly abstract features in an automatic fashion.

However, the adoption of DL approaches for many applications in computational biology has been slow due to seemingly inescapable data challenges, such as low volume, high sparsity and large heterogeneity associated with the use of different profiling platforms. Regarding the last point, we note that multi-platforms studies are especially frequent in traditionally data-scarce domains such as proteomics. Although the minimum amount of training data depends on many variables, such as the complexity of the task, or the type of noise and data distribution, it is generally accepted that one roughly needs at least 10 times more training samples than parameters. As an example, the 2015 computer vision’s model that beat humans at the task of image classification (Alom *et al.*, 2018) exploited large datasets such as ImageNet (Russakovsky et al., 2015) and iNaturalist (Van Horn et al., 2018), consisting of 1 120 000 and 579 184 images to classify 1001 and 5089 classes respectively. In contrast, the largest proteomic cohorts comprise a few hundred of samples, and each sample requires gigabytes of storage instead of the megabytes typically required for images (Liang et al., 2020). An additional level of complexity is presented by the intrinsic nature of tandem mass spectrometry, where each sample consists of a precursor profile (MS1) and multiple precursor fragment profiles (MS2) and all spectra need to be jointly analyzed to quantitatively characterize a sample.

Despite these challenges, successful applications of deep learning in the field of proteomics have been developed. For instance, DeepNovo–DIA (Tran et al., 2019) captures precursor and fragment ions to identify novel peptides in human antibodies and antigens. However, with DeepNovo–DIA only a handful of spectra close to the investigated feature along the retention-time axis are used, somewhat mitigating the aforementioned restrictions.

*Transfer learning for SWATH-MS profile analysis*: Transfer learning is the ability to reuse the knowledge gathered from a learning task in an unrelated and oftentimes completely different task (Pan and Yang, 2010). Humans are extremely good at transfer learning, and, for instance, an English speaker will learn Spanish much faster if she already speaks Italian. In the context of machine learning, transfer learning has been applied by repurposing a model for different tasks than their original target task. The underlying assumption is that if two datasets share a common latent space, a model trained on one dataset can export the data relationships learned to the second dataset (Pan et al., 2008).

In this work, we demonstrate an application of transfer learning to automatically classify tumor versus normal samples using raw SWATH-MS profiles. Specifically, we transform raw MS data into an image format, which enables us to reuse pre-trained DL models for image classification, and later transfer the model to a MS-related classification task. In our implementation, the first transferred layers, which have learned to recognize basic image features, are left unchanged and only their output is coupled to different classifier algorithms. Even with such a naive implementation, we can achieve a remarkable classification performance of 0.876 AUC. Our results suggest that applying deep learning and transfer learning techniques might pave the way to a broader usage of raw mass spectrometry data in real-time diagnosis.

## 2 Materials and methods

The goal of this work is to demonstrate that it is possible to process raw liquid chromatography/mass spectrometry data acquired by SWATH-MS as images, encode them as feature vectors and use them for classification purposes using standard machine learning approaches. In this section, we describe the main components of our approach.

### 2.1 Data

From the Prostate Cancer Outcomes Cohort (ProCOC, ‘PPP1 project’) (Umbehr et al., 2008), 554 tissue biopsies, including both benign and tumor regions for each patient, were sampled from 277 prostate cancer patients. The inclusion of technical replicates resulted in a total of 913 samples considered in this work. Of these, 455 samples are from healthy prostatic tissue and 458 samples are from different types of malignant tumors. Each individual sample of the raw data consists of one precursor profile (MS1) and 100 multiple precursor fragment profiles (MS2) obtained with PCT-SWATH (Guo *et al.*, 2015). A summary of data acquisition and processing to obtain the protein expression vectors can be found in Supplementary Information, Supplementary Section S1. Data acquisition protocols are described in Charmpi *et al.* (2020).

### 2.2 Gold standards

To quantify the accuracy of the tested models, we use protein expression vectors where peptides were quantified using targeted data analysis with OpenSWATH (Röst et al., 2014) as gold standard. Peptide quantification exploits prior knowledge about the chromatographic and mass spectrometric properties using curated and annotated collections of peptide spectra (Ludwig *et al.*, 2018).

We note that the gold standards are not exempt from biases themselves. For instance, while high performance and accuracy are typically observed for high-abundance proteins, systematic deviations from the expected values are observed for low-intensity signals. The deviations depend on the different physicochemical properties of the peptides and are ubiquitous among the different label-free quantification proteomic software tools. Similarly, all software tools depend on the reliable identification of specific peptides. For this, either tandem MS (MS/MS) libraries coupled with statistical methods to separate true from false matches, or ‘pseudo’-MS/MS spectra that do not require an assay library can be used. In both cases, incorrect peptide identification results in inaccurate protein identification and quantification. Despite these shortcomings, reliable and accurate protein quantification is typically achieved with (SWATH)-MS software methods (Navarro *et al.*, 2016).

In a typical MS-experiment, many peptides cannot be quantified in a run for technical or biological reasons, which results in a large number of missing values. We denote this initial dataset after some minimal processing *peptides2* (Table 1). To partially overcome this challenge, missing values can be imputed using technical replicates, resulting in the *peptides3* dataset. Peptides that still present missing values in some samples putation, or display constant values across all samples are excluded from further analysis. Finally, the most informative peptides are selected, resulting in the *peptides4* dataset. Table 1 describes the different processing steps applied to each dataset. A more detailed description of the different processing steps can be found in Zhu et al. (2021).

**Table 1.** Summary of peptide vector post-processing

Name	Processing steps
*peptides2*	Log2 transformation and quantile normalization of samples.
*peptides3*	Imputation of missing values on *peptides2* using technical replicates.
*peptides4*	Batch normalization over different machine runs performed on *peptides3* using ComBat Stein *et al.* (2015).
*proteins*	Selection of only the top 3 peptides per protein (over all samples).
	Imputation of missing values with a linear regression.
	The strongest intensity of proteotypic peptides are adopted as protein intensity.
	

*Note*: During peptide processing, four different intermediate datasets are generated. We test the accuracy of our model on the *peptides3*, *peptides4* and *proteins* datasets.

Each processing step described in Table 1 results in a different number of retained features, as detailed in Table 2. Due to the elimination of samples with missing values, the size of the *peptides3* and *peptides4* datasets is significantly decreased, from around sixteen thousand peptides features to 1207. The last row of Table 1 resulted in 265 quantified proteins, which we use throughout this work as gold standard.

**Table 2.** Peptide and proteomic feature vectors

Dataset	Number of features	Number of retained features
*peptides2*	16 644	0
*peptides3*	16 644	1207
*peptides4*	16 104	1207
*proteins*	2103	265

*Note*: Summary of the number of initial features and retained features after pre-processing as described in Table 1. As there are no features without any missing value in at least one sample before imputation in the original dataset (*peptides2*), all features are eliminated, resulting in zero retained features. We investigate the influence of the different post-processing pipelines in the model’s classification accuracy in Section 3.2.

### 2.3 Mass spectra profiles as images

The output format of different mass spectrometers is vendor-specific, however, most formats can be converted to mzXML format (Pedrioli *et al.*, 2004) with the ProteoWizard software (Chambers *et al.*, 2012). We further modified the Proteowizard software to transform the mzXML input into images of predefined size. According to the SWATH-MS acquisition scheme (Gillet *et al.*, 2012b), MS1 spectra range from 400 to 1249 *m*/*z*, while for MS2 they range from 0 to 2000 *m*/*z*. Hence, we extended the size of MS1 spectra by adding black padding pixels in the ranges 0–400 and 1250–2000 *m*/*z*. With this modification, all spectra cover the range 0–2000 *m*/*z* and the same pipeline can be applied to both MS1 and MS2 raw data formats. Regarding the RT, most of the experiments have a similar duration, and hence, we scaled the time dimension to a uniform time duration. At this point, we define a grid over the *m*/*z* spectrum and the RT, and aggregate all individual pixel values over each bin by computing the average. In a typical SWATH experiment, small changes in the RT of a liquid chromatography method are observed between different runs. These shifts might be due to fluctuations in the concentration of the organic solvent, changes in the flow rate, temperature or even depend on the molecular weight of the peptides. While we do not explicitly model this variability in this work, we expect that the binning in the RT dimension partially decreases the stochasticity on the RT dimension. Finally, we consider two different image sizes, i.e. 512× 512 and 2048×2048 bins over the *m*/*z* and RT dimensions. The sizes were selected to be a power of two to increase the computational efficiency in using the cache, and to be not extremely far from the dimensions of the network architecture used for feature extraction.

### 2.4 Feature vectors

To process raw MS images, we first need to *encode* them, i.e. transform them into numerical vectors that can be further processed. For such an encoding, we use pretrained deep convolutional neural networks developed to classify natural images (Cui *et al.*, 2018; He *et al.*, 2016; Howard *et al.*, 2017; Liu *et al.*, 2018; Real *et al.*, 2019; Sandler *et al.*, 2018; Szegedy *et al.*, 2015; 2017; Zoph *et al*., 2018). In these models, the first layers ingest and analyze pixel information, while the following layers sequentially transform the information into numeric vectors (Alain and Bengio, 2016) to generate the so-called off-the-shelf features. Off-the-shelf features have been shown to be very powerful descriptors of an input image (Sharif Razavian *et al.*, 2014), even for images belonging to classes not included in the training set. Hence, according to the transfer learning philosophy, we generate off-the-shelf MS features from each MS image, and use these features as input for a classifier, which then makes a prediction about the sample. Importantly, no further fine-tuning is performed on the extracted features due to the small number of MS samples available for analysis, which precludes extensive retraining or fine-tuning.

We use modules a selection of publicly available models from TensorflowHub as encoder, see Table 3. The table also presents an overview of the vector encodings for all considered models. We briefly describe the architectural families and respective naming conventions of trained variants included in our study:

**Table 3.** Vector encodings overview

Encoder name [ref]	Input	Output	**Retained features** (for 512×512) in	
			*ms1_only*	*ms1_and_ms2*
resnet_v2_101 (He *et al.*, 2016)	224×224×3	2048	942 (45%)	120 577 (58%)
resnet_v2_50 (He *et al.*, 2016)	224×224×3	2048	1145 (55%)	132 459 (64%)
resnet_v2_152 (He *et al.*, 2016)	224×224×3	2048	1570 (76%)	164 260 (79%)
nasnet_large (Zoph *et al.*, 2018)	331×331×3	4032	3671 (91%)	365 296 (89%)
inception_resnet_v2 (Szegedy *et al.*, 2017)	299×299×3	1536	1536 (100%)	155 107 (99%)
inception_v3_imagenet (Szegedy *et al.*, 2016)	299×299×3	2048	2045 (99%)	206 835 (99%)
inception_v2 (Szegedy *et al.*, 2016)	224×224×3	1024	1018 (99%)	103 418 (99%)
inception_v3_inaturalist (Cui *et al.*, 2018)	299×299×3	2048	2044 (99%)	206 725 (99%)
amoebanet_a_n18_f448 (Real *et al.*, 2019)	331×331×3	7168	5114 (71%)	594 543 (82%)
nasnet_mobile (Zoph *et al.*, 2018)	224×224×3	1056	618 (58%)	88 492 (82%)
inception_v1 (Szegedy *et al.*, 2015)	224×224×3	1024	922 (90%)	102 641 (99%)
pnasnet_large (Liu *et al.*, 2018)	331×331×3	4320	4050 (93%)	427 948 (98%)
mobilenet_v2_050_224 (Sandler *et al.*, 2018)	224×224×3	1280	1178 (92%)	118 715 (91%)
mobilenet_v2_075_224 (Sandler *et al.*, 2018)	224×224×3	1280	1181 (92%)	116 858 (90%)
mobilenet_v1_050_224 (Howard *et al.*, 2017)	224×224×3	512	491 (95%)	50 680 (98%)
mobilenet_v2_100_128 (Sandler *et al.*, 2018)	128×128×3	1280	988 (77%)	102 634 (79%)
mobilenet_v2_075_96 (Sandler *et al.*, 2018)	96×96×3	1280	787 (61%)	92 169 (71%)
mobilenet_v1_025_224 (Howard *et al.*, 2017)	224×224×3	256	249 (97%)	25 026 (96%)
mobilenet_v1_050_128 (Howard *et al.*, 2017)	128×128×3	512	446 (87%)	46 829 (90%)

*Note*: Characteristics of image to feature vector encoders available from https://tfhub.dev/, i.e. image input resolution and output vector size. For any given dataset, constant features over all samples were removed. Feature retention is reported for grid size 512×512 and is virtually the same for grid size 2048×2048.

- **NASNet**s are model architectures found with the Neural Architecture Search (NAS), an automated machine learning structure for training new neural networks. NASNet begins with an overall predefined architecture, but optimizes blocks by a reinforcement learning search method (Zoph *et al.*, 2018). Also exploiting NASNet, AmoebaNet-A (Real *et al.*, 2019) is a convolutional neural network, where the architecture of its convolutional cells (or layers) has been found by an evolutionary architecture search in the NASNet search space. For pnasnet_large (Liu *et al.*, 2018), sequential model-based optimization strategies were used to search for structures in order of increasing complexity, while simultaneously learning a surrogate model to guide the search through structure space. The mobile variant is designed for a constrained computational setting and has a reduced number of parameters and multiply accumulate operations.
- **ResNet** is based on deep residual networks, a family of extremely deep architectures that utilize skip connections, i.e. shortcuts to jump over some layers. Residual networks have shown compelling accuracy and good convergence behavior (He *et al.*, 2016). The last digit in the encoder names in Table 3 represents the number of layers.
- **Inception** is a family of deep convolutional neural network architectures with improved utilization of the computing resources achieved through careful design to enable increased network depth and width, while keeping the computational budget constant (Szegedy *et al.*, 2015, 2016, 2017). Inception-v2 (Szegedy *et al.*, 2016) uses batch normalization at each mini-batch training (Ioffe and Szegedy, 2015), allowing the use of a much higher learning rate and making the network more robust to initialization choices. Inception_v3_inaturalist (Cui *et al.*, 2018) exploits a training scheme that uses higher image resolution and deals with the long-tailed distribution of training data. The knowledge learned from large scale datasets is transferred via fine-tuning to smaller, domain-specific datasets.
- **MobileNet**s are designed to run on mobile devices and primarily use depth-wise separable convolutions to reduce the computational burden (Howard *et al.*, 2017). MobileNetV2 is based on an inverted residual structure where the input and output of the residual block are thin linear bottleneck layers (Sandler*et al.*, 2018). Model variant names include, as percentage, a multiplier for the depth in the convolutional layers to control model size and lastly the image input size that affects inference speed.

All the feature encoders were trained on ImageNet (Russakovsky *et al.*, 2015), an extensive image database where images are organized according to word concepts, e.g. cat, bird, flower, etc. The only exception is inception_v3_inaturalist which was trained on the iNaturalist dataset (Van Horn *et al.*, 2018), a dataset of animal pictures. The encoders were developed to ingest color images of predefined sizes. To apply the encoders to the MS images, the MS images are processed in two ways. First, we triplicate the gray-scale channel as rgb channels. Secondly, we resize the images to fit the required encoder input size (often 224×224, see Table 3) using bilinear scaling, Tensorflow’s default resize method. No further pre-processing is performed on the raw data.

Encoding raw MS images as vectors allows us to concatenate the one MS1 and 100 MS2 images (spectra) associated with the same sample into a single vector, which enables us to compare the classification performance of models trained uniquely using MS1 spectra (*ms1_only*) against models exploiting all spectra (*ms1_and_ms2*).

However, a downside of including both modalities is that the number of features is significantly increased. To keep the number of features manageable, considering the number of samples available, we eliminate constant features, i.e. features exhibiting a zero standard deviation across all samples. Almost all off-the-shelf feature representations result in some constant features, with the single exception of inception_resnet_v2, which retains all 1536 features. On the other extreme, resnet_v2_101 retains the smallest percentage of features. The percentage of retained features for each encoder is shown in Table 3.

### 2.5 Evaluation through classification performance

Each one of the tested encoders described in Section 2.4 transforms LC-MS/MS data acquired from a single sample into a numerical vector. At this point, we can define various options for MS image resolution, type of encoder and type of spectral data included, resulting in several tabular datasets from the same raw MS data. Each dataset, including the peptide and proteomic gold standards defined in Section 2.2, can be further processed by a downstream machine learning algorithm, hence allowing us to compare the predictive power of the datasets and to investigate the impact of the different options considered when creating the dataset. As we do not wish to limit our analysis to a particular predictive model, we extend our comparison to several of the most frequently used classifiers based on different theoretical foundations, including logistic regression, support vector classification (SVC), random forest and gradient boosted trees (XGBoost), see Table 4.

**Table 4.** Classification algorithms and hyperparameter values tested during optimization

Classifier	Parameter	Values
Logistic regression (LG)	C	0.1, 1, 10, 100
Support vector machine (SVC)	C	0.1, 1, 10, 100
	kernel	’linear’, ’poly’, ’rbf’
Random forest (RF)	n_estimators	100, 500
Gradient boosted trees (XGBoost)	n_estimators	100, 500

*Note*: ‘C’ is a regularization parameter of inverse strength. ‘linear’, ‘poly’ and ‘rbf’ kernel functions refer to the linear, polynomial and radial basis function, respectively. ‘n_estimators’ is the number of trees in the forest. Classifiers are implemented using scikit-learn (Pedregosa *et al.*, 2011), with the exception of XGBoost (Chen and Guestrin, 2016).

For all derived datasets, we apply the following pipeline. First, all random seeds are fixed for comparability and reproducibility. Next, a random stratified (meaning the ratio of the classes is kept constant) test set comprising 30% of samples is excluded from training. The retained features are scaled to the range [0, 1] (per feature) on the training set, and the learned transformation reapplied to the test set. To optimize the hyperparameters, we perform a shallow grid search of hyperparameters (see Table 4) via internal six-fold cross–validation and two repeats. The optimization results in an independent set of hyperparameters for each combination of MS image resolution, encoder, *ms1_only* versus *ms1_and_ms2* and classification algorithm. The classifier is trained on the full training set by using the hyperparameters with the best mean test performance measured by AUC. Finally, the trained classifier is evaluated on the test set. We reiterate that, as we implemented a random stratified data split, the ratio of the classes is kept constant in the training and test datasets. The Python code used for the entire pipeline(including data splits, hyperparameter optimization and analysis) can be accessed at https://ibm.biz/mstransc.

## 3 Results

We present a comparison of the phenotype classification performances obtained with different proteomic feature vectors (see Section 2.2) and off-the-shelf features (see Section 2.4). We investigate different metrics to quantify the classification performance, including Brier loss, Log loss, Accuracy, F1 score, Youden’s index, Recall, Precision and Specificity. Here, we report only the area under the receiver operating characteristic curve (AUC), while performances based on the additional metrics are available in Supplementary Information.

### 3.1 Encoders performance

**Fig. 1.**
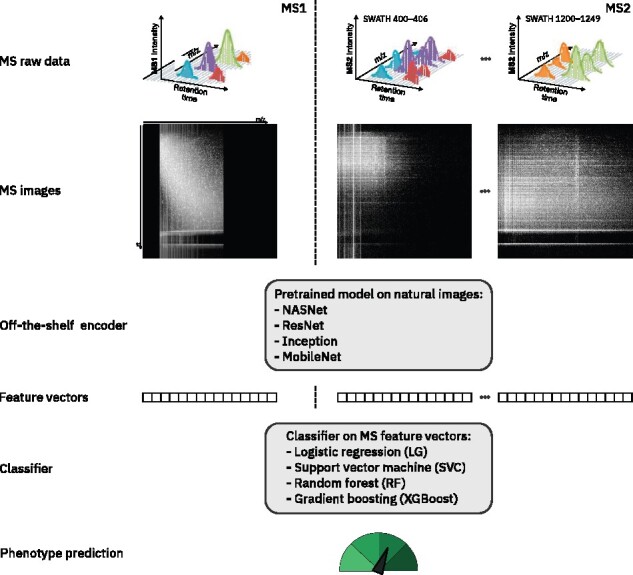
Sample representations in the workflow. Mass spectra of MS1 and MS2 scans, illustrated in the top row as 3D views adopted from Ludwig et al.(2018, CC BY 4.0), constitute the raw data for a single sample. Each individual scan is processed to an image representation by rasterizing along the retention time (*rt*) and mass charge ratio (*m/z*) axes. The MS images are then resized and the gray-scale channel triplicated to match the input dimensions of the chosen image–to–vector encoder. The MS images are then given as an input to different publicly available models pretrained for natural image classification. Each model transforms the raw MS images into *feature vectors*, i.e. numerical vectors that encode the information contained in the image. The resulting vectors can consist of encodings from MS1 only (*ms1_only*), or optionally the concatenation of both MS1 and all MS2 encodings (*ms1_and_ms2*). Using the feature vectors, a classifier is trained on the training set to predict the phenotype, i.e. cancer or normal sample, and evaluated on the test set. We compare performance between multiple combinations of generated image resolution, encoder and classification algorithm

Figure 1shows the evaluation score of all representations combining both MS1 and MS2 spectra (*ms1_and_ms2*) and the gold standards. To facilitate comparisons, for each encoder, we average across classifiers and image resolutions and report only the median AUC achieved using both M1 and M2 spectra (*ms1_and_ms2*).

The ResNet architectures achieve superior performance with both best median (0.849 AUC by resnet_v2_101) and best single result (0.876 AUC by resnet_v2_50), followed by Inception and NASNet architectures that range from 0.827 to 0.777 median AUC. The worst performance is achieved by mobilenet encoders, with the lowest median AUC of 0.623 by mobilenet_v1_050_128. However, even in this case, the AUC is significantly above 0.5 (random prediction). See Table 5 for statistics on all encoders and gold standards. As a group, MobileNet models exhibit the weakest performance and show large variability for different choices of classifier and image resolution.

**Table 5.** Summary of classification performances for encoders and gold standards

	median AUC		mean AUC		*σ* AUC		architecture
Available input	MS1/2	MS1	MS1/2	MS1	MS1/2	MS1	
Encoder							
Proteins	0.951	NaN	0.947	NaN	0.010	NaN	Proteomics
peptides3	0.951	NaN	0.948	NaN	0.012	NaN	Proteomics
peptides4	0.947	NaN	0.945	NaN	0.012	NaN	Proteomics
resnet_v2_101	0.849	0.759	0.837	0.764	0.036	0.029	ResNet
resnet_v2_50	0.834	0.784	0.826	0.783	0.029	0.013	ResNet
resnet_v2_152	0.832	0.746	0.824	0.747	0.045	0.025	ResNet
nasnet_large	0.827	0.749	0.816	0.757	0.029	0.025	NASNet
inception_resnet_v2	0.820	0.737	0.817	0.740	0.043	0.025	Inception, ResNet
inception_v3_imagenet	0.811	0.770	0.814	0.766	0.029	0.018	Inception
inception_v2	0.806	0.745	0.795	0.735	0.030	0.022	Inception
inception_v3_inaturalist	0.795	0.732	0.800	0.727	0.023	0.022	Inception
amoebanet_a_n18_f448	0.793	0.733	0.788	0.730	0.034	0.023	NASNet
nasnet_mobile	0.792	0.714	0.792	0.710	0.028	0.015	NASNet
inception_v1	0.789	0.717	0.777	0.719	0.035	0.021	Inception
pnasnet_large	0.777	0.748	0.773	0.746	0.023	0.017	NASNet
mobilenet_v2_050_224	0.765	0.622	0.758	0.609	0.039	0.031	MobileNet
mobilenet_v2_075_224	0.737	0.524	0.717	0.520	0.055	0.032	MobileNet
mobilenet_v1_050_224	0.704	0.582	0.686	0.583	0.084	0.044	MobileNet
mobilenet_v2_100_128	0.687	0.485	0.674	0.486	0.050	0.018	MobileNet
mobilenet_v2_075_96	0.666	0.530	0.670	0.534	0.057	0.027	MobileNet
mobilenet_v1_025_224	0.656	0.636	0.643	0.637	0.043	0.040	MobileNet
mobilenet_v1_050_128	0.623	0.512	0.616	0.512	0.036	0.030	MobileNet

*Note*: For each feature encoding module, median, mean and standard deviation (*σ*) of the classification performance AUC values over the different classifiers are reported. For each statistic, the input of MS image features concatenated (*ms1_and_ms2*, in the table MS1/2) is neighbored by MS1 image features only (*ms1_only*, in the table MS1) for comparison.

**Fig. 2.**
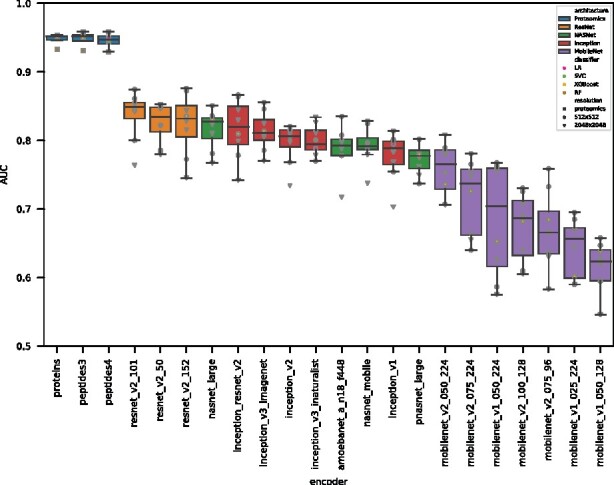
Encoding module and choice of classifier drive classification performance. Publicly available modules—trained to classify natural images—were used to encode off-the-shelf feature vectors. Exceptions to this are the gold standard datasets *proteins*, *peptides3* and *peptides4*, which were obtained using a curated proteomics analysis pipeline. Classification performance, measured by AUC, is reported in order of descending median AUC for different classifiers and two resolutions of MS images (rasterized spectra). Here, we only report results obtained using concatenated feature vectors encoded from MS1 and all MS2 images (*ms1_and_ms2*). As observed in the figure, the main driver of performance is the encoding of features. Different off-the-shelf features achieve results ranging from 0.623 up to 0.849 median AUC, while gold standard features reached 0.951 median AUC. The variance over results from different classifiers is much larger for off-the-shelf features compared to the gold standard features

**Fig. 3.**
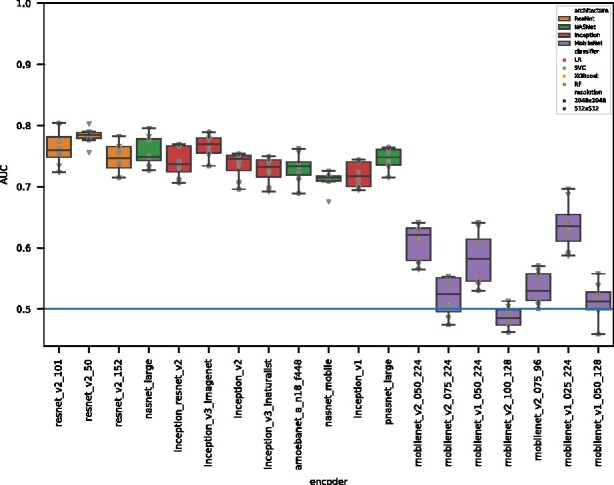
Classification performance of *ms1_only* off-the-shelf features. Depicted is the same plot as in Figure 2, but with *ms1_only* encodings instead of *ms1_and_ms2*. The order of encoders is identical, with the peptide and protein datasets missing as these cannot be compared to a case where MS2 information is excluded. While the classification performance of *ms1_only* encodings is generally lower compared to *ms1_and_ms2*, there is a pronounced drop in performance for mobilenets, with some models performing even worse than random (AUC below 0.5). Different off-the-shelf features achieve results ranging from 0.485 up to 0.784 median AUC

Similar patterns in terms of the ranked encoder family’s performance are obtained for the off-the-shelf encoders when only the MS1 image is used, see Figure 2. ResNet encoders still perform best, closely followed by NASNet and Inception encoders. Reflecting this similarity, the Spearman’s rank correlation between performances achieved with MS1 and MS2 or only MS1 is 0.808, see Figure 3.

Similar results are obtained if we average across classifiers and resolutions for each encoder by computing the median, although the Spearman’s rank correlation is now slightly higher at 0.875 (see Table 5). Overall, it is quite remarkable that we were able to create high-quality features on a vastly different domain and downstream task without any fine-tuning.

### 3.2 Gold standards performance

As expected, the classification performance using the gold standards, as defined in Section 2.4, is very good. The best individual result was achieved for the *peptides4* dataset (AUC 0.959 achieved with XGBoost). When the results across classifiers are averaged, all gold standard datasets achieve a very high AUC. The *proteins* and *peptides3* representations are virtually indistinguishable, see Table 5, despite the *proteins* dataset including a much smaller number of features—265 features versus 1207 features in the *peptides3* and *peptides4* datasets, see Table 1. This suggests that the removal of non-proteotypic peptides and selection of top 3 peptides per protein does indeed preserve most of the biological information contained in the *peptides4* dataset.

Interestingly, there are only very minor differences between the performances achieved by the three gold standards. All three evaluated gold standards achieve very good performances, with only a small decrease in standard deviation on the *proteins* dataset, see Table 5 and Figure 1. One might have expected that the *proteins* gold standard performs better than the peptides-based gold standards, as it integrates biological knowledge about proteotypic peptides and penalizes peptides less highly expressed and, hence, more likely to be randomly profiled. However, there are two additional considerations to keep in mind. First, *peptides3* and *peptides4* incorporate a larger number of features, some of which might not be very informative. This might make the classifiers harder to train and result in a larger variability across hyperparameters. Second, as stated in Section 2.2, peptides that still present missing values after imputation are eliminated from the *peptides3* dataset. These peptides are not likely to be missing because of technical reasons, but might quite possibly be biologically meaningful peptides that differentiate samples. By removing them, one might be depleting the more processed datasets, i.e. *peptides4* and *proteins*, of important biological signals. For the most processed dataset, i.e. the *proteins* dataset, this factor might counteract the gain originating from the integration of biological knowledge and reduction of the number of features, resulting in a similar performance to the *peptides3* and *peptides4* datasets, and only a slightly reduced standard deviation.

### 3.3 Classifier agreement

Classifiers trained on the gold standard datasets perform very similarly, see Figure 1 and Supplementary Table S1. Logistic regression, SVC and XGBoost models achieve almost indistinguishable performances, only the random forest classifiers showed a weaker performance.

Regarding the classifiers trained on the off-the-shelf encodings, SVC and logistic regression consistently achieve better performances, while random forest performs equally well than XGBoost. However, when using *ms1_and_ms2* (very long vectors), random forest performs worse as XGBoost, with a mean difference of 0.043 AUC across all encodings and resolutions. As random forest is methodically close to XGBoost—both methods are based on decision tree algorithms, with XGBoost benefiting from a more advanced training—the observed difference between both methods is expected to be alleviated by further hyperparameter optimization.

### 3.4 Classification performance when using only MS1 compared to using both MS1 and MS2 spectra

**Fig. 4.**
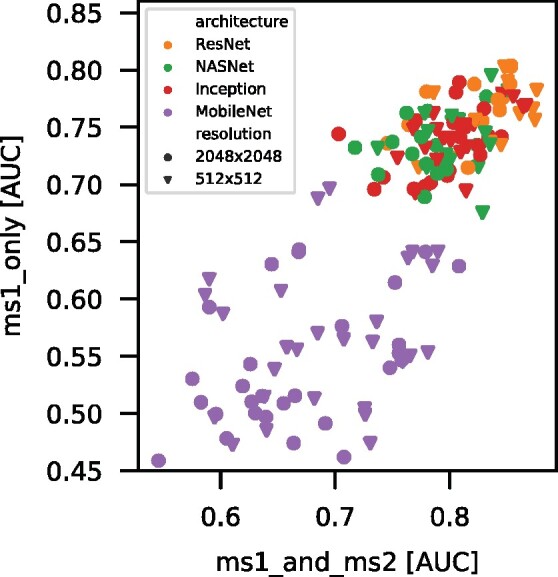
Correlation of *ms1_only* and *ms1_and_ms2* AUC. The figure shows the Spearman’s rank correlation of the AUC obtained using *ms1_only* versus *ms1_and_ms2*. Although the absolute values differ, the ordering of points is highly similar in both dimensions. The overall Spearman’s rank correlation is 0.81. The median values per encoder exhibit a Spearman’s rank correlation of 0.88

We also investigate whether the inclusion of MS2 images improves the classification performance, and if so, by how much. To investigate this question, as described in Section 2.4, we created two different datasets, *ms1_only*, which includes only the MS1 raw vector, and *ms1_and_ms2*, which consists of a vector concatenation of the single MS1 and 100 MS2 spectra associated with the same sample. Figure 4A clearly demonstrates that a higher performance is achieved when both MS1 and MS2 spectra are included. A one sided paired t-test with null hypothesis of ms1_and_ms2⩽ms1_only was rejected under α= 0.001 (*P*-value of 2.61 $\times 10^{-38}$). The distribution of differences in AUC (see Fig. 4B) shows a small benefit (mean delta of 0.085 AUC) of including MS2 features. The distribution shows a smaller peak with larger deltas (slightly violating the normality assumption) that stems solely from results of mobilenets.

A possible downside of including all MS modalities is a sharp increase in the number of features—a single MS1 image might require hundreds or thousands of features depending on the model, while the concatenation of all images results in hundreds of thousands features. The significantly larger number of features might enable the encoding of more detailed information about *m*/*z* ranges in MS1 spectra. This might boost performance, although the large number of features might increase the need of having larger datasets or lead to overfitting when small datasets are used. Appropriate regularization techniques, or feature pre-selection by, for instance, removing features with low signal-to-noise ratios, might alleviate overfitting. To enhance applicability in a clinical setting, however, it would be desirable to limit data pre-processing and feature selection and/or engineering to an absolute minimum. Hence, while waiting for the next generation of larger proteomics datasets to come, smaller models might be preferable.

### 3.5 MS image resolution

The resolution of the MS images (as described in Section 2.3) also influences the performances of the different classifiers. We find a small but significant difference in the evaluation scores that favors the 512×512 resolution versus 2048×2048, as seen in Figure 4C. Indeed, a one sided paired t-test with the null hypothesis ${\text{AUC}}_{512x512}⩽{\text{AUC}}_{2048x2048}$ was rejected with a level of significance α=0.001. For these settings, the mean difference in AUCs is 0.016, which leads to rejection of the null hypothesis (*P*-value of 9.22 $\times 10^{-11}$). Note that images, regardless of the original resolutions, are resized according to each encoder’s input size requirements (see Table 3), most often to 224×224.

## 4 Discussion

Quantitative proteomics enable the unbiased and faithful characterization of molecular phenotypes. A recent benchmark of several computational workflow tools for the analysis of SWATH-MS data has highlighted the convergent identification and reliable quantification performance of all tools Navarro *et al.* (2016). However, these workflows require laborious and carefully fine-tuned pre- and post-data processing, and various challenges hinder their broad application in a clinical setting. For instance, SWATH-MS data analysis relies on targeted data extraction strategies, which query the acquired fragment ion maps using a priori information obtained from spectral libraries to identify and quantify peptides Ludwig *et al.* (2018). While methods have been developed that do not require spectral libraries Ting *et al.* (2017), their analysis is limited to peptides of known sequence. Furthermore, although it achieves good performance, targeted analysis discards a lot of information, e.g. by ignoring proteins not represented in the spectral library or by limiting quantification to a selection of expected peaks. In doing so, subtle differences between related proteoforms, which might be informative to correctly classify a sample, might be discarded early on in the processing pipeline. It is therefore highly desirable to develop alternative approaches that can exploit the whole proteome information and require minimum or no feature selection.

**Fig. 5.**
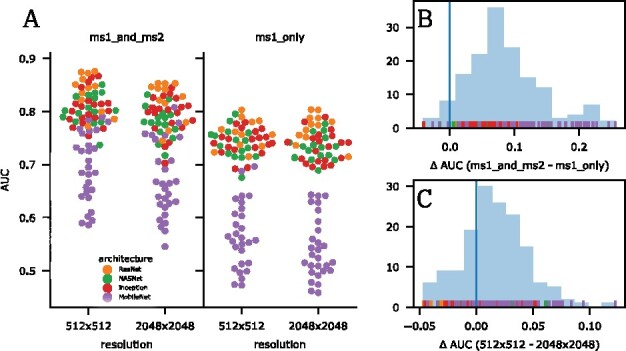
Effects of input features on classification performance. Each encoding module was applied to images resulting from two different resolutions of grids (512×512 and 2048×2048) on the spectra, with some initial resizing to fit the module’s specific input dimensions. Also, features from either only MS1 or MS1 and all MS2 (concatenated) were used as input to train four different classifiers. (A) We observe a clear gain in classification performance when including MS2 features and a slight difference for the change in resolution. (B) The distribution of differences in AUC (*ms1_only*—*ms1_and_ms2*) shows a significant benefit (mean of 0.085 AUC) for including MS2 features. The distribution shows a smaller peak with larger deltas (slightly violating the normality assumption) that stems solely from results of mobilenets. (C) The distribution of differences in AUC for different resolutions shows a significant advantage (mean of 0.016 AUC) of using the smaller grid size that is closer to the module’s typical input size and requires less downsizing in the encoding step

Deep learning approaches have an extraordinary capacity to automatically learn abstract discriminative features directly from raw data. At the same time, transfer learning is known to be able to achieve very good performances in complex tasks where little data is available, as is often the case in biological data cohorts. Combining the two, we have investigated whether deep learning models for natural image classification (off-the-shelf encoders) can be exploited to produce informative feature vectors directly from raw SWATH-MS spectral profiles. Although raw MS images (Fig. 5) are extremely different from natural images, we were able to achieve very good accuracies for the task of classifying tumor versus normal prostate tissue biopsies.

As gold standard for comparison, we used protein quantification derived from the standard SWATH analysis pipeline tuned by domain experts. While the gold standards achieves better classification results than the off-the-shelf encoders, the generation of peptide and protein datasets from MS raw images requires highly supervised pipelines supplemented with prior knowledge from peptide libraries. Contrary to the careful data processing and model fine-tuning of MS-workflows, our approach does not use any prior knowledge nor does it require any fine-tuning.

As one MS1 and 100 MS2 profiles are obtained from a single sample, we investigated two alternative ways of ingesting raw SWATH-MS data, one based on the sole analysis of MS1 images, and another one that jointly exploits MS1 and MS2 images. Our results show that the inclusion of both MS1 and MS2 information boosts the classification performance of all models, although at the price of significantly increasing the number of features. Therefore, when smaller MS-cohorts are used to train the classifiers, the use of only MS1 spectra might be a preferred strategy to prevent overfitting. However, our cross-validation analysis highlighted how regularization and the removal of features with low signal-to-noise ratio results in extremely reliable and robust performance, confirming that overfitting can be mitigated even when dealing with a limited number of samples.

Several improvements might further increase the performance of our model and will be investigated in future work. In terms of biological variability, multiple run alignments might help account for retention time variation between runs, and noise filtering and batch correction might lead to additional accuracy gains in the models exploiting off-the-shelf features. Regarding model improvements, our current model does not reflect the relationship of the different MS2 swathes and MS1. Alternative ways of encoding raw MS data to reflect their dependencies will be investigated in future work. More importantly, from the engineering point of view, our method drastically downsizes the original raw SWATH-MS images to relatively small image sizes. This process is likely to result in accuracy loss, e.g. by binning together peaks that could otherwise be informative for classifying samples. Ingesting the raw data in an uncompressed fashion would be very promising, although this would increase the required number of training samples drastically. However, with the rising popularity of mass spectrometry, larger size cohorts are expected to become available in the near future. Larger cohorts will undoubtedly enable training new or fine-tuning the transferred models, resulting in increased model performance.

Another interesting avenue to explore would be leveraging existing unlabeled spectra to learn MS-specific features. Namely, instead of using pretrained models for natural image classification, autoencoders (Kramer, 1991; Vincent *et al.*, 2010) could be trained on existing large raw MS datasets from databases such as PRIDE (Perez-Riverol *et al.*, 2019) and Peptide Atlas (Desiere *et al.*, 2006). Using MS-specific architectures, this strategy would enable the transformation of raw MS images into feature vectors using unsupervised deep learning techniques. The most fascinating aspect of this approach is the possibility of addressing two challenges at once: data scarcity, since we can learn from spectra generated with different instruments and species; and the need of learning features that are more specific to the MS images. The results obtained in this work using models pretrained on natural images testifies how promising such methods can be.

As an application example, consider the scenario where the cancerous tissue expresses a proteoform with N-terminal truncation compared to the healthy tissue, i.e. some peptides are missing in the cancer proteoform due to the pathophysiology of the disease. Such peptides are expected to be highly discriminative and are expected to be captured on MS images. However, in the conventional SWATH analysis, they are likely to be eliminated somewhere along the processing path unless specifically searched for. By contrast, learning from MS images does not pre-select features, and hence, can identify subtle differences across samples. Furthermore, one can easily envision enhanced models that search for such small differences, e.g. by exploiting interpretability methods (Dhurandhar *et al.*, 2018) to identify the key features that underlie the biological similarities and differences. This is in stark contrast to conventional analysis that can only reveal peptide differences once they have been hypothezised.

To conclude, our results show that networks trained on natural images can generalize to the analysis of MS images surprisingly well. This opens the door to the development of future models trained on larger cohorts of MS images, potentially accelerating the development of deep learning models for proteomics applications in both research and clinical settings.

## Funding

This project received funding from the European Union s Horizon 2020 Research and Innovation Program (668858 and 826121), the Australian Cancer Research Foundation and Cancer Institute NSW (2017/TPG001).

*Conflict of Interest*: none declared.

## Supplementary Material

btab311_Supplementary_Data
