# Correction to: On the feasibility of deep learning applications using raw mass spectrometry data

**DOI:** 10.1093/bioinformatics/btad106

**Published:** 2023-03-10

This is a correction to: Joris Cadow, Matteo Manica, Roland Mathis, Roger R. Reddel, Phillip J. Robinson, Peter J. Wild, Peter G. Hains, Natasha Lucas, Qing Zhong, Tiannan Guo, Ruedi Aebersold, María Rodríguez Martínez, On the feasibility of deep learning applications using raw mass spectrometry data, *Bioinformatics*, Volume 37, Issue Supplement_1, July 2021, Pages i245–i253, https://doi.org/10.1093/bioinformatics/btab311

In the originally published version of this manuscript the following authors were inadvertently omitted:

- Roger R. Reddel
- Phillip J. Robinson
- Peter J. Wild
- Peter G. Hains
- Natasha Lucas
- Qing Zhong

The ‘Funding’ section of the article has been updated to reflect the correct author list with the addition of funding from “the Australian Cancer Research Foundation and Cancer Institute NSW (2017/TPG001).”

The ‘Availability and implementation’ section of the papers has also been updated from:

“The open source code used to generate the results from MS images is available on GitHub: https://ibm.biz/mstransc. The raw MS data underlying this article cannot be shared publicly for the privacy of individuals that participated in the study. Processed data including the MS images, their encodings, classification labels and results can be accessed at the following link: https://ibm.box.com/v/mstc-supplementary.”

To:

“The open source code used to generate the results from MS images is available on GitHub: https://ibm.biz/mstransc. The data, including the MS images, their encodings, classification labels and results, can be accessed at the following link: https://ibm.ent.box.com/v/mstc-supplementary.”

These errors have been corrected.

