# Supplementary material for: On the feasibility of deep learning applications using raw mass spectrometry data
Source: Bioinformatics. 2021 Jul 12;37(Suppl 1):i245–53. doi: 10.1093/bioinformatics/btab311 (PMC8275322; doi:10.1093/bioinformatics/btab311)
Supplement: btab311_Supplementary_Data [file btab311_supplementary_data.pdf]

Supplementary Information – On the Feasibility of Deep Learning Applications Using Raw Mass Spectrometry Data

1 Proteomic data acquisition

The following paragraph provides experimental details for proteomic data acquisition of data used in this work, while also trying to summarize the general process of LC-MS/MS for the unfamiliar reader.

The tissue samples are processed into peptides and analyzed with SWATH-MS as described previously [2]. The proteins are extracted from tissue samples using 6M urea and 2M thiourea, then digested into peptides using trypsin and Lys-C. The tissue lysis and protein digestion are assisted by pressure cycling technology (PCT). The peptides mixture is separated by liquid chromatography (LC) over 90 min gradient where the different peptides are eluted in Gaussian distribution around a characteristic retention time (rt).

The continuously eluted peptides are fed into a tandem mass spectrometry assembly (MS/MS, Sciex TripleTOF 6600 mass spectrometry). In a mass spectrometer the input molecules are ionized and accelerated. The ions separate according their characteristic mass charge ratio (m/z) and signal intensity proportional to the number of ions can be recorded (scanned). Depending on the spectrometers accuracy, the ions are accelerated more or less consistently, such as that detection of many of identical ions can be assumed to be Gaussian-distributed over small range along the m/z axis. Such a scan is recorded in the first mass spectrometer (MS1, sometimes LC-MS Map), from the peptides –here called precursors– currently eluted from the LC column. Each scan takes only a short time and consumes only a fraction of any precursor. Alternatively to recording in MS1, the separation of precursors can be used to isolate some of them within fixed windows along the m/z axis, called swathes. Directed towards a second mass spectrometer, the isolated precursor ions of a given swath are fragmented into smaller "fragment ions" and recorded (MS2).

With the SWATH-MS acquisition scheme as described by [1], the MS1 range 400-1249 m/z (containing most peptides) is covered by sequentially isolating variable sized swathes towards the second mass spectrometer for 0.1 second each and scanned along the MS2 range 0-2000 m/z. The specific swath sizes are shown in

Figure S1, with larger swathes in ranges known to contain fewer precursors.

A cycle over all 100 MS2 scans, including a first MS1 scan, where precursors are neither filtered nor fragmented, takes 10.1 seconds. Over the duration of the assay the spectra per cycle are stacked for MS1 and for MS2 per respective swath, each sample results in 101 2–dimensional (rt, m/z) spectral profiles with peaks of signals constituting the raw data.

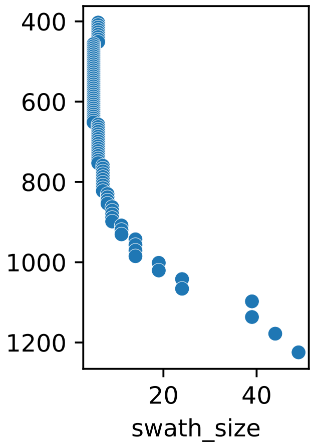

Figure S1: **Swathes** along the m/z axis in MS1 (vertical axis), each resulting in an MS2 scan, vary in size.

2 Results for all models

AUC score for all the models are reported in Table S1.

		AUC							
	available input	ms1_and_ms2				ms1_only			
	classifier	LR	RF	SVC	XGBoost	LR	RF	SVC	XGBoost
encoder	resolution								
proteins	proteomics	**0.953**	0.933	0.952	0.951	NaN	NaN	NaN	NaN
peptides3	proteomics	**0.959**	0.931	0.953	0.95	NaN	NaN	NaN	NaN
peptides4	proteomics	0.95	0.929	0.944	**0.959**	NaN	NaN	NaN	NaN
resnet_v2_101	2048x2048	0.853	0.764	0.853	0.842	0.804	0.752	0.803	0.774
	512x512	0.861	0.8	**0.874**	0.845	0.762	0.723	0.756	0.735
resnet_v2_50	2048x2048	0.847	0.78	**0.853**	0.821	0.776	0.781	0.788	0.788
	512x512	0.851	0.786	0.848	0.822	0.79	0.78	0.803	0.756
resnet_v2_152	2048x2048	0.843	0.745	0.828	0.816	0.765	0.736	0.756	0.715
	512x512	0.872	0.773	**0.876**	0.835	0.766	0.715	0.782	0.737
nasnet_large	2048x2048	0.832	0.767	0.81	0.824	0.777	0.727	0.751	0.734
	512x512	**0.851**	0.781	0.836	0.831	0.782	0.747	0.795	0.745
inception_resnet_v2	2048x2048	0.845	0.742	0.83	0.799	0.742	0.706	0.766	0.712
	512x512	0.864	0.778	**0.866**	0.809	0.768	0.732	0.769	0.728
inception_v3_imagenet	2048x2048	0.805	0.77	0.808	0.825	0.781	0.756	0.789	0.734
	512x512	0.846	0.786	**0.855**	0.813	0.779	0.762	0.777	0.752
inception_v2	2048x2048	0.809	0.734	0.803	0.798	0.742	0.696	0.754	0.708
	512x512	0.816	0.768	**0.820**	0.815	0.751	0.748	0.752	0.733
inception_v3_inaturalist	2048x2048	**0.834**	0.779	0.826	0.811	0.742	0.699	0.726	0.75
	512x512	0.788	0.77	0.801	0.789	0.748	0.692	0.738	0.722
amoebanet_a_n18_f448	2048x2048	0.788	0.778	0.717	0.797	0.734	0.689	0.732	0.713
	512x512	0.808	0.8	0.778	**0.835**	0.757	0.721	0.762	0.735
nasnet_mobile	2048x2048	0.794	0.738	0.779	0.789	0.717	0.709	0.718	0.709
	512x512	0.825	0.79	0.797	**0.828**	0.715	0.713	0.726	0.676
inception_v1	2048x2048	0.798	0.703	0.783	0.769	0.711	0.744	0.702	0.696
	512x512	0.795	0.754	0.801	**0.814**	0.74	0.723	0.741	0.694
pnasnet_large	2048x2048	0.78	0.75	0.762	0.775	0.764	0.737	0.763	0.742
	512x512	**0.802**	0.737	**0.802**	0.78	0.755	0.732	0.76	0.715
mobilenet_v2_050_224	2048x2048	**0.808**	0.706	0.779	0.752	0.629	0.576	0.641	0.614
	512x512	0.789	0.707	0.785	0.736	0.641	0.565	0.629	0.58
mobilenet_v2_075_224	2048x2048	0.756	0.664	0.748	0.655	0.553	0.474	0.54	0.509
	512x512	0.765	0.64	**0.781**	0.726	0.55	0.485	0.553	0.499
mobilenet_v1_050_224	2048x2048	0.756	0.575	0.758	0.626	0.56	0.53	0.547	0.543
	512x512	0.763	0.586	**0.767**	0.653	0.636	0.603	0.641	0.607
mobilenet_v2_100_128	2048x2048	0.707	0.605	0.691	0.64	0.462	0.478	0.491	0.497
	512x512	**0.730**	0.61	0.726	0.682	0.474	0.473	0.504	0.513
mobilenet_v2_075_96	2048x2048	0.665	0.583	0.636	0.63	0.515	0.51	0.515	0.5
	512x512	0.733	0.667	**0.759**	0.685	0.562	0.556	0.545	0.57
mobilenet_v1_025_224	2048x2048	0.668	0.59	0.668	0.644	0.641	0.593	0.643	0.631
	512x512	0.695	0.59	0.685	0.602	**0.696**	0.617	0.688	0.587
mobilenet_v1_050_128	2048x2048	0.619	0.546	0.627	0.596	0.524	0.459	0.51	0.5
	512x512	**0.658**	0.594	0.647	0.638	0.558	0.497	0.539	0.515

Table S1: **All individual results of classification performance** measured in AUC. The best result per encoder is bold.

References

[1] Ludovic C Gillet, Pedro Navarro, Stephen Tate, Hannes Röst, Nathalie Selevsek, Lukas Reiter, Ron Bonner, and Ruedi Aebersold. Targeted data extraction of the MS/MS spectra generated by data-independent acquisition: a new concept for consistent and accurate proteome analysis. *Molecular & cellular proteomics : MCP*, 11(6):O111.016717, jun 2012. ISSN 1535-9484. doi: 10.1074/mcp.O111.016717.

URL http://www.ncbi.nlm.nih.gov/pubmed/22261725http://www.pubmedcentral.nih.gov/articlerender.fcgi?artid=PMC3433915. 1

[2] Tiannan Guo, Petri Kouvonen, Ching Chiek Koh, Ludovic C Gillet, Witold E Wolski, Hannes L Röst, George Rosenberger, Ben C Collins, Lorenz C Blum, Silke Gillessen, Markus Joerger, Wolfram Jochum, and Ruedi Aebersold. Rapid mass spectrometric conversion of tissue biopsy samples into permanent quantitative digital proteome maps. *Nature medicine*, 21(4):407, 2015. doi: 10.1038/NM.3807. URL https://www.ncbi.nlm.nih.gov/pmc/articles/PMC4390165/. 1
